# The effectiveness and safety of repetitive transcranial magnetic stimulation on spasticity after upper motor neuron injury: A systematic review and meta-analysis

**DOI:** 10.3389/fncir.2022.973561

**Published:** 2022-11-08

**Authors:** Jin Fan, Hui Fu, Xiaolong Xie, Dongling Zhong, Yuxi Li, Xiaobo Liu, Huiling Zhang, Jun Zhang, Jiaxi Huang, Juan Li, Rongjiang Jin, Zhong Zheng

**Affiliations:** ^1^School of Health Preservation and Rehabilitation, Chengdu University of Traditional Chinese Medicine, Chengdu, China; ^2^Mental Health Center, West China Hospital, West China School of Medicine, Sichuan University, Chengdu, China; ^3^Department of Rehabilitation Medicine, West China Second University Hospital, Sichuan University, Chengdu, China; ^4^Department of Rehabilitation Medicine, The Affiliated Meishan Hospital of Chengdu University of Traditional Chinese Medicine, Meishan, China

**Keywords:** upper motor neuron injury, repetitive transcranial magnetic stimulation, spasticity, systematic review, meta-analysis

## Abstract

To systematically evaluate the effectiveness and safety of repetitive transcranial magnetic stimulation (rTMS) on spasticity after upper motor neuron (UMN) injury. Eight electronic databases were searched from inception to August 6, 2022. Randomized controlled trials (RCTs) investigating the effectiveness and safety of rTMS on spasticity after UMN injury were retrieved. Two reviewers independently screened studies, extracted data, and assessed the risk of bias. Review Manager 5.3 and Stata 14.0 software were used to synthesize data. The certainty of the evidence was appraised with the Grade of Recommendation, Assessment, Development and Evaluation tool. Forty-two studies with a total of 2,108 patients were included. The results of meta-analysis revealed that, compared with control group, rTMS could significantly decrease scores of the Modified Ashworth Scale (MAS) in patients with UMN injury. The subgroup analysis discovered that rTMS effectively decreased the MAS scores in patients with stroke. Meanwhile, rTMS treatment > 10 sessions has better effect and rTMS could decrease the MAS scores of upper limb. Thirty-three patients complained of twitching facial muscles, headache and dizziness, etc. In summary, rTMS could be recommended as an effective and safe therapy to relieve spasticity in patients with UMN injury. However, due to high heterogeneity and limited RCTs, this conclusion should be treated with caution.

## Introduction

The spasticity refers to abnormal increase of muscle tone, which is associated with upper motor neuron (UMN) injury occurring in stroke, spinal cord injury (SCI), cerebral palsy (CP), multiple sclerosis (MS), and others (Dietz and Sinkjaer, 2012; Posteraro et al., 2018). After UMN injury, owing to loss of supraspinal inhibition, bulbospinal pathways become hyperexcitable, the presynaptic inhibition of muscle spindle afferents reduce and muscular tone increase (Li S. et al., 2021). Spasticity is characterized by a velocity-dependent increase in tonic stretch reflexes (muscle tone) with exaggerated tendon jerk (Feldman et al., 1980). Roughly, the prevalence of spasticity reaches to 42.6% (Harb and Kishner, 2022) in stroke patients, and 73.5% of patients with SCI may accompany spasticity (Strom et al., 2022). Moreover, approximately 80% of patients with MS (Arroyo González, 2018) and 69.8% children with CP (Pulgar et al., 2019) experience spasticity.

Spasticity could restrict joint movement, cause low dexterity of movement, abnormal limb postures, and pain (Naro et al., 2017). The spasticity reduces patients’ ability to undertake activities of daily living, such as walking, eating, and bathing (Ward, 2012). Patients with long-term spasticity usually accompany with depression, anxiety, bipolar disorder, and other mood disorders (Chen et al., 2013; Kes et al., 2013). The common pharmacological treatments for spasticity are oral muscle relaxants (Yelnik et al., 2009; Sommerfeld et al., 2012), intrathecal baclofen (Ertzgaard et al., 2017; Creamer et al., 2018), and botulinum neurotoxin injections (Chen et al., 2020; Harriss et al., 2021). However, the efficacy of antispastic drugs is limited, and long-term medication may cause undesirable side effects, such as drowsiness, cognitive impairment, and muscle weakness (Langhorne et al., 2011; Turner-Stokes et al., 2015). Consequently, it is necessary to find an effective and safe therapy to alleviate spasticity.

Repetitive transcranial magnetic stimulation (rTMS) is a method that delivers TMS pulses in trains with a constant frequency and intensity to induce changes in brain activity (Nardone et al., 2020). During rTMS treatment, a coil is placed on head, when a current is passing through a coil, a magnetic field can be generated (Roth et al., 1991). Magnetic field evokes a current which has impact on cortical excitability. Modulation of cortical excitability could induce cortical plastic changes (Nowak et al., 2009). Neuroplasticity refers to the ability of the nervous system to adjust activity after injury (Puderbaugh and Emmady, 2022). rTMS has been reported to be able to trigger neuroplasticity and potentiate synaptic transmission (Iglesias, 2020; Cantone et al., 2021). It is inferred that the anti-spastic effect of rTMS may be associated with the neuroplasticity modulation. Gottlieb et al. found that rTMS could reduce MAS scores in stroke patients and regulate neuronal plasticity (Gottlieb et al., 2021). Another study revealed that rTMS reduced spasticity in incomplete SCI patients by increasing synaptic transmission (de Araujo et al., 2017). Therefore, rTMS is a promising therapy to promote neuroplasticity and ameliorate spasticity.

Transcranial magnetic stimulation has been widely used to treat spasticity after UMN injury including stroke (Rastgoo et al., 2016), SCI (Kumru et al., 2013), CP (Rajak et al., 2019), and MS (San et al., 2019). Previous systematic reviews and meta-analyses (Gao et al., 2018; McIntyre et al., 2018; Xu P. et al., 2021; Wang et al., 2022) have been conducted to evaluate the effect of rTMS in patients with stroke and SCI. Gao et al. found that rTMS could improve the spasticity in patients with incomplete SCI (Gao et al., 2018). Wang et al. concluded that rTMS had a significant effect to relieve spasticity in patients with stroke (Wang et al., 2022). While the other two systematic reviews and meta-analyses (McIntyre et al., 2018; Xu P. et al., 2021) reported that rTMS was not effective to improve spasticity after stroke. Furthermore, the optimal protocols of rTMS (e.g., intensity, frequency, pulses, treatment site, number of sessions etc.) for spasticity remains to be investigated. Recently, several clinical trials of rTMS on spasticity after UMN injury have been conducted. We intended to conduct the systematic review and meta-analysis to update the current evidence of rTMS for spasticity after UMN injury and to explore optimal protocols of rTMS.

## Methods

We conducted this systematic review and meta-analysis strictly following the A Measurement Tool to Assess Systematic Reviews (AMSTAR 2.0) (Shea et al., 2017) and reported according to the Preferred Reporting Items for Systematic reviews and Meta-Analysis 2020 (PRISMA 2020) statement guidelines (Page et al., 2021). The protocol of this study has been registered in the international prospective register of systematic reviews (PROSPERO, https://www.crd.york.ac.uk/prospero/display_record.php?ID=CRD42020213173). The registration number is CRD42020213173. The completed PRISMA 2020 checklist is shown in Supplementary Appendix 1.

### Inclusion criteria

#### Type of studies

Randomized controlled trials (RCTs) and cross-over RCTs (Hui et al., 2015) that investigated the effect of rTMS for spasticity after UMN injury were included. The language was limited to Chinese or English.

#### Type of participants

Participants with spasticity after UMN injury (stroke, CP, MS, SCI, etc.) were included (Posteraro et al., 2018). The spasticity was defined that Modified Ashworth Scale (MAS) was greater than 0 (Balci, 2018), the Brunnstrom stage was greater than I or author reported spasticity. There were no restrictions on age, gender, race, or nation.

#### Type of interventions

The interventions included rTMS or rTMS combined with conventional rehabilitation (CR) training (physiotherapy, occupational therapy, orthotics, etc.).

#### Type of comparators

The comparators involved sham rTMS, CR or sham rTMS plus CR.

#### Outcome measurements

The primary outcome was MAS scores. The secondary outcomes included H_max_/M_max_ ratio, F-wave latency, Fugl-Meyer-Assessment (FMA) and Barthel Index (BI), Hamilton anxiety scale (HAMA), Hamilton depression scale (HAMD). In addition, rTMS related adverse events (headache, seizures, hearing impairment, etc.) were assessed as safety measurements.

### Exclusion criteria

Studies were excluded if they met one of the following criteria: (1) factorial RCTs (Montgomery et al., 2003), N of 1 RCT (Ulbrich-Zurni et al., 2018) or cluster RCTs (Ribeiro et al., 2018); (2) full text were unavailable through various approaches; (3) duplications; (4) the data cannot be extracted; (5) other patterns of TMS, such as deep TMS, paired associative stimulation; (6) other non-invasive brain stimulation techniques, such as transcranial direct current stimulation (tDCS) and electrical stimulation alone (Brihmat et al., 2022).

### Search strategy

We systematically searched China National Knowledge Infrastructure, the Chinese Science and Technology Periodical Database, Wanfang database, China Biology Medicine, PubMed, Embase, the Cochrane Library, and Web of Science from their inception to August 6, 2022. The medical subject headings (MeSH) and free terms were combined using Boolean logic operators. The full search strategies which were tailored according to the characteristic of the above databases are listed in Supplementary Appendix 2. We manually searched gray literature, reference lists of identified studies for possible relevant literatures. Additionally, the Chinese Clinical Trial Registry and ClinicalTrials.gov were searched and the experts were consulted for eligible RCTs.

### Studies selection

All the retrieved records were imported into Endnote (X9), then the duplicated records were removed. After that, two reviewers (Jin Fan and Hui Fu) independently screened titles and abstracts. Then, the rest records in full text were thoroughly reviewed according to eligible criteria. Any discrepancy was resolved by discussion or consultation with a third independent reviewer (Juan Li).

### Data extraction

A standardized data extraction form was designed in advance. We piloted data extraction with three eligible studies, and evaluated the intraclass correlation coefficient (ICC) to achieve reliability in extraction. Two researchers (Yuxi Li and Xiaobo Liu) independently extracted the following data: (i) study information: the first author, year of publication, type of study; (ii) participant characteristics: sample size, gender, age, types of UMN injury, course of disease; (iii) intervention details: intervention, coil type, pulse, frequency, intensity, site, sessions of treatment; (iv) study outcomes: indicators of spasticity (MAS, Hmax/Mmax ration, F-wave latency, etc.) and other relevant outcomes; (v) information related to risk of bias. The original authors were contacted for missing data if necessary. For multi-arm RCTs, the comparison with inferior effect size was pooled to obtain more conservative results. After extraction, cross-check was performed to ensure no mistakes. Disagreements were arbitrated by a third reviewer (Rongjiang Jin).

### Assessment of risk of bias

The revised Cochrane risk of bias tool for individually randomized, parallel group trials (ROB 2.0) tool was used to assess the risk of bias (Yang et al., 2017). Two independent reviewers (Xiaolong Xie and Huiling Zhang) studied the ROB 2.0, then the trained reviewers pre-assessed three eligible studies and calculated the ICC. After achieving good reliability in the risk of bias assessments, we performed formal evaluation.

### Data analysis

The ICC was used to determine the level of reliability between reviewers. The classification of ICCs is: excellent reliability (ICC > 0.90), good reliability (ICC = 0.76–0.90), moderate reliability (ICC = 0.50–0.75), and poor reliability (ICC < 0.50) (Grgic et al., 2022). SPSS (version 25.0) was used to calculate ICC. For the cross-over RCTs, we extracted and analyzed the data at the first intervention phase. The change of MAS was used to estimate the effect size. The mean difference (MD) was used to analyze continuous outcomes with the same unit, otherwise standardized MD (SMD) was calculated. Heterogeneity of included studies was assessed using the Cochrane *Q* test and was quantified by the estimated *I*^2^ statistic. A fixed-effect model was applied if heterogeneity was acceptable (*I*^2^ ≤ 50%, *P* ≥ 0.1). Otherwise, a random-effect model was chosen. If outcomes could not be quantitatively analyzed, we narratively described these results. For all outcome variables, two-tailed *P*-values < 0.05 were considered statistically significant. Meta-analysis was conducted with the Review Manager (RevMan, version 5.3.5) and Stata (version 14.0) software.

### Subgroup analysis

We conducted subgroup analysis based on the types of UMN injury (stroke, CP, SCI, MS), the frequency of rTMS (low frequency, high frequency), the intensity of rTMS [≤ 90% Motor threshold (MT), > 90% MT], the total sessions of rTMS (≤10, >10), the assessment position of the MAS (upper limb, lower limb).

### Sensitivity analysis

The sensitivity analysis was conducted by deleting each study one by one to verify the robustness of the results.

### Publication bias

The funnel plot was used to describe possible publication bias when ≥ 10 studies included in the analysis. In addition, the *Begg’s* test and *Egger’s* test were also used.

### The certainty of evidence

We used the Grading of Recommendations Assessment, Development and Evaluation (GRADE) approach (Guyatt et al., 2011) to appraise the certainty of evidence. The GRADE comprises five items: risk of bias, inconsistency, indirectness, imprecision, and publication bias (Atkins et al., 2004). To ensure a reliability in evaluation of GRADE, we pre-assessed three samples and calculated the ICC as well. The certainty of evidence of each outcome was considered as high, moderate, low, or very low by two independent reviewers (Yuxi Li and Dongling Zhong). GRADEpro (Version 3.6) software was adopted to summarize the findings.

## Result

### Selection of eligible studies

**FIGURE 1:**
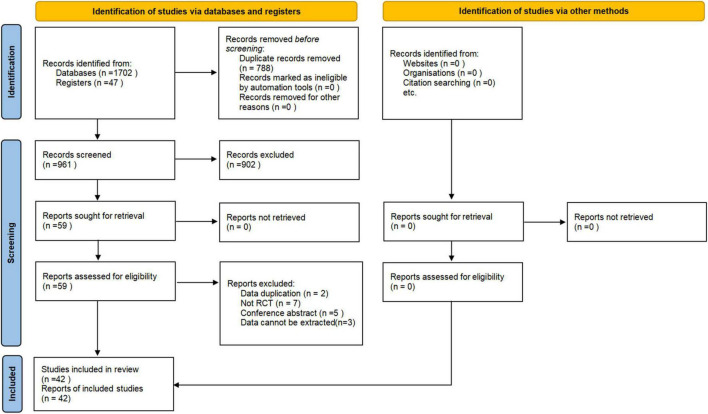
PRISMA flowchart.

A total of 1,749 records were retrieved through electronic search. After removing duplicates, the title and abstract of the rest records were screened. Then, 59 articles were remained for scrutinization with the full texts. Seventeen studies were excluded, and the reasons for exclusion are listed in Supplementary Appendix 3. Eventually, 42 eligible RCTs with a total of 2,108 patients (Valle et al., 2007; Kumru et al., 2010, 2016; Benito et al., 2012; Nardone et al., 2014; Liao et al., 2015; Yan, 2015; Bao and Liu, 2016; Askin et al., 2017; Li, 2017; Ozkeskin et al., 2017; Sun et al., 2017; Wu, 2017; Chervyakov et al., 2018; Kong, 2018; Liu et al., 2018, 2019; Qin et al., 2018a,b; Tao and Wei, 2018; Watanabe et al., 2018; Xiao, 2018; Zhang, 2018; Dos Santos et al., 2019; Korzhova et al., 2019; Liu, 2019; Luo, 2020; Qi, 2020; Yuan, 2020; Zhang et al., 2020; Chen et al., 2021; Gottlieb et al., 2021; Liang et al., 2021; Mendonca et al., 2021; Xu R. et al., 2021; Yang, 2021; Zhao, 2021; Cheng et al., 2022; Xia et al., 2022a,b; Yang and Yang, 2022; Zang et al., 2022) were included. The PRISMA flow diagram is shown in Figure 1.

### The characteristics of included studies

The included studies were published from 2007 to 2022. The age of patients with CP, SCI, stroke severally ranged from 1.54 to 14.4 years old, 20.33 to 65.18 years old, and 35.51 to 77.33 years old. The sample size of included trials varied from 9 to 240. Thirteen articles (Valle et al., 2007; Kumru et al., 2010, 2016; Benito et al., 2012; Nardone et al., 2014; Askin et al., 2017; Ozkeskin et al., 2017; Chervyakov et al., 2018; Watanabe et al., 2018; Dos Santos et al., 2019; Korzhova et al., 2019; Gottlieb et al., 2021; Mendonca et al., 2021) were published in English and twenty-nine articles (Liao et al., 2015; Yan, 2015; Bao and Liu, 2016; Li, 2017; Sun et al., 2017; Wu, 2017; Kong, 2018; Liu et al., 2018, 2019; Qin et al., 2018a,b; Tao and Wei, 2018; Xiao, 2018; Zhang, 2018; Liu, 2019; Luo, 2020; Qi, 2020; Yuan, 2020; Zhang et al., 2020; Chen et al., 2021; Liang et al., 2021; Xu R. et al., 2021; Yang, 2021; Zhao, 2021; Cheng et al., 2022; Xia et al., 2022a,b; Yang and Yang, 2022; Zang et al., 2022) in Chinese. Twenty-eight studies (Liao et al., 2015; Askin et al., 2017; Li, 2017; Ozkeskin et al., 2017; Sun et al., 2017; Wu, 2017; Chervyakov et al., 2018; Kong, 2018; Liu et al., 2018, 2019; Qin et al., 2018a,b; Tao and Wei, 2018; Watanabe et al., 2018; Xiao, 2018; Dos Santos et al., 2019; Liu, 2019; Luo, 2020; Yuan, 2020; Zhang et al., 2020; Chen et al., 2021; Gottlieb et al., 2021; Xu R. et al., 2021; Yang, 2021; Zhao, 2021; Cheng et al., 2022; Xia et al., 2022a,b) involved stroke, seven studies (Kumru et al., 2010, 2016; Benito et al., 2012; Nardone et al., 2014; Liang et al., 2021; Mendonca et al., 2021; Yang and Yang, 2022) related to SCI, six studies (Valle et al., 2007; Yan, 2015; Bao and Liu, 2016; Zhang, 2018; Qi, 2020; Zang et al., 2022) focused on CP, and one (Korzhova et al., 2019) about MS. The frequency of rTMS varied from 1 to 20 Hz. Seventeen studies (Kumru et al., 2010, 2016; Nardone et al., 2014; Li, 2017; Chervyakov et al., 2018; Qin et al., 2018b; Xiao, 2018; Zhang, 2018; Korzhova et al., 2019; Luo, 2020; Qi, 2020; Liang et al., 2021; Mendonca et al., 2021; Xia et al., 2022a,b; Yang and Yang, 2022; Zang et al., 2022) adopted high-frequency stimulation, and the remaining studies used low-frequency stimulation. The intensity of rTMS was from 20%MT to 120%MT. Twenty-three studies (Liao et al., 2015; Askin et al., 2017; Li, 2017; Ozkeskin et al., 2017; Sun et al., 2017; Wu, 2017; Chervyakov et al., 2018; Kong, 2018; Liu et al., 2018; Qin et al., 2018b; Tao and Wei, 2018; Watanabe et al., 2018; Zhang, 2018; Dos Santos et al., 2019; Korzhova et al., 2019; Liu, 2019; Yuan, 2020; Chen et al., 2021; Xu R. et al., 2021; Yang, 2021; Zhao, 2021; Cheng et al., 2022; Xia et al., 2022a) stimulated unaffected hemisphere, nine studies (Li, 2017; Chervyakov et al., 2018; Qin et al., 2018a,b; Xiao, 2018; Liu et al., 2019; Luo, 2020; Xia et al., 2022a,b) treated affected hemisphere, four studies (Yan, 2015; Bao and Liu, 2016; Chervyakov et al., 2018; Zang et al., 2022) involved bilateral rTMS, while eleven studies (Valle et al., 2007; Kumru et al., 2010, 2016; Benito et al., 2012; Nardone et al., 2014; Qi, 2020; Zhang et al., 2020; Gottlieb et al., 2021; Liang et al., 2021; Mendonca et al., 2021; Yang and Yang, 2022) did not specify the stimulation side. Among the included studies, there were comparisons of rTMS plus CR versus sham rTMS plus CR, rTMS plus CR versus CR, rTMS versus sham rTMS, and rTMS versus CR. The characteristics of the included studies are shown in Table 1.

**TABLE 1:** Characteristics of the included studies.

Author, year	Type of study	Subjects	Course of disease (month)	Sample size	Female/Male	Age	Experi-mental group	Control group	Coil type	Fre-quency/Intensity	Dura-tion of one session	Number of pulses	Stimu-lated site	The sessions of rTMS	Evaluate position	The indi-cators of spas-ticity	Adverse effects
Chervyakov et al., 2018	RCT	Stroke	E1: 5.1 ± 4.8 E2: 5.8 ± 4.6 E3: 7.37 ± 5.9 C: 7.9 ± 8.4	E1: 11 E2: 13 E3: 8 C: 10	26/16	E: 58.5 ± 10.7 C: 61.4 ± 11.4	rTMS+ CR	Sham rTMS+ CR	E: F8C	E1: 1 Hz/ 100%RMT E2: 10 Hz/ 80%RMT E3: 10 Hz/ 80%RMT + 1 Hz/ 100%RMT C: 10 Hz/ 80%RMT	E1: 20 min E2: 10 min E3: 30 min	NI	E1: unaffected side E2: affected side E3: bilateral side C: bilateral side	E1: 10 sessions E2: 10 sessions E3: 10 sessions C: 10 sessions	Upper limb	MAS	NI
Valle et al., 2007	RCT	Cerebral palsy	NI	E1: 6 E2: 5 C: 6	9/8	E1: 9.8 ± 4.6 E2: 9.8 ± 3.6 C: 8 ± 1.89	rTMS+ CR	Sham rTMS+ CR	E: F8C	E1: 1 Hz/ 90%RMT E2: 5 Hz/ 90%RMT	NI	E1: 1,500 pulses E2: 1,500 pulses	NI	NI	Upper limb	MAS	No serious adverse effects
Askin et al., 2017	RCT	Stroke	E: 28.35 ± 15.34 C: 24.35 ± 15.39	E: 20 C: 20	11/29	E: 56.75 ± 11.46 C: 58.80 ± 12.02	rTMS+ CR	CR	E: F8C	E: 1 Hz/ 90%RMT	E: 20 min	E: 1,200 pulses	E: unaffected side	E: 10 sessions	Upper limb	MAS	No serious adverse effects
Bao and Liu, 2016	RCT	Cerebral palsy	NI	E: 22 C: 23	25/20	E: 3.00 ± 1.09 C: 3.05 ± 1.51	rTMS+ CR	CR	E: circular coil	E: 1 Hz/ 30%MT	NI	E: 600 pulses	E: bilateral side	E: 10 sessions	Upper limb	MAS	NI
Benito et al., 2012	RCT	Spinal cord injury	E: 8.57 ± 2.82 C: 6.8 ± 3.22	E: 7 C: 10	4/13	E: 38.43 ± 14.12 C: 36.5 ± 13.22	rTMS+ CR	Sham rTMS+ CR	E: double cone coil C: double cone disconnected connected F8C	E: 20 Hz/ 90%RMT C: NI	E: 20 min	E: 1,800 pulses	NI	E: 15 sessions C: 15 sessions	Lower limb	MAS	Facial muscle twitching (6/10)
Chen et al., 2021	RCT	Stroke	E: 2.00 ± 1.34 C: 2.17 ± 11.10	E: 30 C: 30	20/40	E: 64.13 ± 13.20 C: 61.37 ± 11.90	rTMS+ CR	Sham rTMS+ CR	E: F8C	E: 1 Hz/ 90%RMT C: 1 Hz/ 90%RMT	E: 20 min	E: 1,200 pulses	E: unaffected side C: unaffected side	E: 20 sessions C: 20 sessions	Upper limb	MAS Hmax/Mmax ratio	No serious adverse effects
Cheng et al., 2022	RCT	Stroke	NI	E: 120 C: 120	76/164	E: 61.58 ± 4.06 C: 61.75 ± 3.97	rTMS + CR	CR	NI	E: 1 Hz/110%MT	NI	E: 1,200 pulses	E: unaffected side	E: 40 sessions	Upper limb	MAS	NI
Gottlieb et al., 2021	RCT	Stroke	NI	E: 14 C: 14	12/16	E: 63.93 ± 10.91 C: 62.43 ± 11.46	rTMS + CR	Sham rTMS + CR	E: F8C	E: 1 Hz/100%RMT	E: 30 min	E: 1,200 pulses	NI	E: 10 sessions C: 10 sessions	Upper limb	MAS	Mild discomfort: headache (4/28), pain in contralateral hand (1/28)
Kong, 2018	RCT	Stroke	E: 1.84 ± 0.61 C: 1.89 ± 0.67	20/20	23/17	E: 50.40 ± 8.40 C: 52.25 ± 8.14	rTMS + CR	Sham rTMS + CR	E: F8C	E: 1 Hz/100%MT C: 1 Hz/20%MT	E: 20 min	E: 800 pulses	E: unaffected side C: unaffected side	E: 20 sessions C: 20 sessions	Upper limb	MAS	No serious adverse effects
Korzhova et al., 2019	RCT	Multiple sclerosis	NI	E: 12 C: 10	9/13	NI	rTMS + CR	Sham rTMS + CR	E: F8C	E: 20 Hz/80%MT	E: 20 min	E: 1,600 pulses	NI	E: 10 sessions C: 10 sessions	Lower limb	MAS	NI
Kumru et al., 2010	RCT	Spinal cord injury	E: 5.64 ± 3.37 C: 5.14 ± 3.39	E: 14 C: 7	3/18	E: 33.93 ± 13.60 C: 41.29 ± 18.51	rTMS	Sham rTMS	E: double cone coil C: double cone disconnected connected F8C	E: 20 Hz/90%RMT	E: 20 min	E: 1,600 pulses	NI	E: 25 sessions C: 25 sessions	Lower limb	MAS Hmax/Mmax ratio	Facial muscle twitching (3/14)
Kumru et al., 2016	RCT	Spinal Cord Injury	E: 2.80 ± 1.52 C: 2.84 ± 1.43	E: 15 C: 16	7/24	E: 46.40 ± 15.50 C: 48.69 ± 16.49	rTMS + CR	Sham rTMS + CR	E: double cone coil C: double cone disconnected connected F8C	E: 20 Hz/90%RMT	E: 20 min	E: 1,800 pulses	NI	E: 20 sessions C: 20 sessions	Lower limb	MAS	Mild discomfort: facial twitching, difficulty to speak (8/15), mild headache (1/15)
Li, 2017	RCT	Stroke	E1: 1.86 ± 1.12 E2: 1.36 ± 1.48 C: 1.58 ± 1.52	E1: 42 E2: 43 C: 42	87/40	E1: 57.87 ± 12.89 E2: 2:54 ± 13.35 C: 53.13 ± 13.72	rTMS	Sham rTMS	E: circular coil	E1: 1 Hz/80%MT E2: 10 Hz/80%MT	E1: 20 min E2: 20 min	E1: 1,000 pulses E2: 1,350 pulses	E1: unaffected side E2: affected side C: affected side	E1: 10 sessions E2: 10 sessions C: 10 sessions	Upper limb	MAS	No serious adverse effects
Liang et al., 2021	RCT	Spinal Cord Injury	E: 1.05 ± 0.54 C: 1.13 ± 0.50	E: 25 C: 25	19/31	E: 40.2 ± 12.6 C: 42.5 ± 16.2	rTMS + CR	Sham rTMS + CR	E: F8C	E: 9 Hz/80%RMT C: 9 Hz	E: 20 min	NI	NI	E: 24 sessions C: 24 sessions	Lower limb	MAS	NI
Liao et al., 2015	RCT	Stroke	E: 1.72 ± 0.24 C: 1.79 ± 0.17	E: 15 C :14	10/19	E: 56.23 ± 10.31 C: 54.93 ± 12.23	rTMS + CR	Sham rTMS + CR	NI	E: 1 Hz, 80%RMT C: 1 Hz, 80%RMT	E: 15 min C: 15 min	E: 1,200 pulses C: 1,200 pulses	E: unaffected side	E: 14 sessions C :14 sessions	Upper and lower limb	MAS	NI
Liu, 2019	RCT	Stroke	E: 2.81 ± 1.27 C: 3.11 ± 1.37	E: 20 C: 20	22/18	E: 61.35 ± 9.43 C: 55.00 ± 11.86	rTMS + CR	CR	NI	E: 1 Hz/120%MT	E: 20 min	E: 1,200 pulses	E: unaffected side	E: 24 sessions	Upper limb	MAS	NI
Liu et al., 2018	RCT	Stroke	E: 4.50 ± 1.90 C: 4.85 ± 2.08	E: 10 C: 13	9/14	E: 56.90 ± 9.02 C: 55.38 ± 8.40	rTMS + CR	CR	NI	E: 1 Hz/90%MT	E: 24 min	E: 1,200 pulses	E: unaffected side	E: 40 sessions	NI	MAS	NI
Liu, 2019	RCT	Stroke	E: 1.51 ± 0.51 C: 2.78 ± 1.70	E: 21 C: 20	18/23	E: 55.43 ± 6.72 C: 58.05 ± 8.48	rTMS + CR	Sham rTMS + CR	E: F8C	E: 10 Hz/80%MT C: 10 Hz/80%MT	E: 20 min	E: 1,500 pulses	E: affected side C: affected side	E: 40 sessions C: 40 sessions	Upper limb	MAS	NI
Luo, 2020	RCT	Stroke	E: 1.78 ± 0.82 C: 1.67 ± 0.87	E: 20 C: 14	8/26	E: 57.25 ± 10.57 C: 53.93 ± 12.9	rTMS + CR	Sham rTMS + CR	E: circular coil	E: 5 Hz/120%RMT C: 5 Hz	E: 20 min	E: 1,000 pulses	E: affected side C: affected side	E: 20 sessions C: 20 sessions	Upper limb	MAS	Nausea, headache (1/34), mild numbness of the scalp and dizziness (1/34)
Ozkeskin et al., 2017	RCT	Stroke	E: 10.45 ± 21.80 C: 24.50 ± 23.88	E: 10 C: 11	8/13	E: 55.70 ± 14.92 C: 64.54 ± 9.38	rTMS + CR	Sham rTMS + CR	NI	E: 1 Hz/90%RMT C: NI	E: 25 min	E: 1,500 pulses	E: unaffected side C: unaffected side	E: 10 sessions C: 10 sessions	Upper limb	MAS	NI
Mendonca et al., 2021	Crossover RCT	Spinal cord injury	E1: 4.73 ± 2.05 E2: 4.73 ± 2.05 C: 4.73 ± 2.05	E1: 11 E2: 11 C: 11	9/24	E1: 35.00 ± 12.12 E2: 35.00 ± 12.12 C: 35.00 ± 12.12	rTMS	Sham rTMS	E: F8C	E1: 1 Hz/90%RMT E2: 10 Hz/90%RMT C: 10 Hz/90%RMT	NI	E1: 1,500 pulses E2: 1,800 pulses	NI	NI	Lower limb	MAS Hmax/Mmax ratio	No serious adverse effects
Nardone et al., 2014	Crossover RCT	Spinal cord injury	NI	E: 4 C: 5	1/8	NI	rTMS	Sham rTMS	E: NI C: double cone disconnected connected F8C	E: 20 Hz/90%RMT	E: 20 min	E: 1,600 pulses	NI	E: 5 sessions C: 5 sessions	Lower limb	MAS Hmax/Mmax ratio H reflex	NI
Qi, 2020	RCT	Cerebral Palsy	E1: 3.25 ± 1.09 E2: 3.40 ± 1.18 C: 3.07 ± 0.96	E1: 15 E2: 15 C: 15	16/29	E1: 4.25 ± 0.66 E2: 4.46 ± 0.61 C: 4.51 ± 0.62	rTMS + CR	Sham rTMS + CR	NI	E1: 1 Hz/90%MT E2: 5 Hz/90%MT C:NI	E1:15 min E2:15 min	E1: 1,200 pulses E2: 1,200 pulses	NI	E1: 60 sessions E2: 60 sessions C: 60 sessions	Lower limb	MAS	No serious adverse effects
Qin et al., 2018a	RCT	Stroke	E1: 2.55 ± 1.57 E2: 2.65 ± 1.90 C: 2.95 ± 1.61	E1: 20 E2: 20 C: 20	36/24	E1: 57.15 ± 9.80 E2: 55.35 ± 6.88 C: 57.30 ± 9.38	rTMS + CR	Sham rTMS + CR	E: F8C	E1: 1 Hz/90%MT E2: 10 Hz/80%MT C: 10 Hz/80%MT	E1: 24 min E2: 20 min	E1: 1,200 pulses E2: 1,500 pulses	E1: unaffected side E2: affected side C: affected side	E1: 40 sessions E2: 40 sessions C: 40 sessions	Upper limb	MAS	NI
Qin et al., 2018b	RCT	Stroke	E: 2.95 ± 1.88 C: 3.10 ± 1.65	E: 20 C: 20	16/24	E: 55.45 ± 9.08 C: 56.75 ± 9.42	rTMS + CR	CR	E: F8C	E: 10 Hz/80%MT	E: 20 min	E: 1,500 pulses	E: affected side	E: 40 sessions	Upper limb	MAS	NI
Dos Santos et al., 2019	RCT	Stroke	E: 47.80 ± 43.20 C: 50.10 ± 27.20	E: 10 C: 10	7/13	E: 52.40 ± 12.00 C: 64.60 ± 6.80	rTMS + CR	Sham rTMS + CR	E: F8C	E: 1 Hz/90%RMT C: NI	NI	E: 1,500 pulses	E: unaffected side	E: 10 sessions C: 10 sessions	Upper limb	MAS Hmax/Mmax ratio	No serious adverse effects
Sun et al., 2017	RCT	Stroke	E: 2.00 ± 1.50 C: 1.80 ± 1.10	E: 20 C: 20	32/8	E: 55.10 ± 8.50 C: 53.50 ± 7.90	rTMS + CR	Sham rTMS + CR	E: circular coil	E: 1 Hz/80%MT	NI	E: 1,200 pulses	E: unaffected side	E: 24 sessions C: 24 sessions	Upper limb	MAS F-wave latency	NI
Tao and Wei, 2018	RCT	Stroke	E: 4.01 ± 2.89 C: 3.58 ± 2.44	E: 24 C: 24	26/22	E: 56.55 ± 13.11 C: 57.33 ± 12.00	rTMS + CR	CR	NI	E: 1 Hz/90%MT	E: 15 min	E: 1,200 pulses	E: unaffected side	NI	NI	MAS	No serious adverse effects
Watanabe et al., 2018	RCT	Stroke	NI	E: 7 C: 6	4/9	NI	rTMS + CR	Sham rTMS + CR	E: F8C	E: 1 Hz/110%RMT C: 80%RMT	NI	E: 1,200 pulses	E: unaffected side C: affected side	E: 10 sessions C: 10 sessions	Upper limb	MAS	NI
Wu, 2017	RCT	Stroke	E: 19.58 ± 6.78 C: 20.04 ± 6.41	E: 24 C: 23	23/24	E: 58.29 ± 7.26 C: 55.83 ± 9.20	rTMS + CR	Sham rTMS + CR	E: F8C	E: 1 Hz/90%MT C: 1 Hz/90%MT	E: 24 min	E: 1,200 pulses	E: unaffected side C: unaffected side	E: 40 sessions C: 40 sessions	NI	MAS	NI
Xia et al., 2022a	RCT	Stroke	E1: 2.61 ± 1.25 E2: 2.55 ± 1.19 C: 2.47 ± 1.13	E1: 18 E2: 18 C: 18	23/31	E1: 70.40 ± 2.10 E2: 69.10 ± 1.90 C: 69.60 ± 1.70	rTMS + CR	CR	NI	E1: 1 Hz/90%MT E2: 10 Hz/110%MT	NI	E: 1,200 pulses	E1: unaffected side E2: affected side	E1: 20 sessions E2: 20 sessions	Upper limb	MAS	Headache (3/54)
Xia et al., 2022b	RCT	Stroke	E: 2.55 ± 1.31 C: 2.41 ± 1.16	E: 40 C: 40	33/47	E: 69.30 ± 1.90 C: 69.90 ± 1.80	rTMS + CR	CR	NI	E: 20 Hz/90%MT	NI	E: 1,400 pulses	E: affected side	E: 20 sessions	Upper limb	MAS	Headache (5/120)
Xiao, 2018	RCT	Stroke	E1: 2.16 ± 0.96 E2: 2.1 ± 1.25 C: 2.34 ± 1.29	E1: 16 E2: 15 C: 17	32/16	E1: 58.63 ± 9.07 E2: 63.73 ± 11.00 C: 58.65 ± 10.84	rTMS + CR	Sham rTMS + CR	E: F8C	E1: 3 Hz/90%RMT E2: 10 Hz/90%RMT C: 10 Hz/90%RMT	E1: 30 min E2: 9 min	E1: 900 pulses E2: 900 pulses	E1: affected side E2: affected side	E1: 10 sessions E2: 10 sessions C: 10 sessions	Upper limb	MAS	NI
Xu R. et al., 2021	RCT	Stroke	E: 7.07 ± 5.24 C: 6.20 ± 3.47	E: 15 C: 15	3/27	E: 47.13 ± 11.62 C: 54.47 ± 11.62	rTMS + CR	Sham rTMS + CR	E: F8C	E: 1 Hz/80%RMT	E: 20 min	E: 1,200 pulses	E: unaffected side C: NI	E: 25 sessions C: 25 sessions	Upper limb	MAS Hmax/Mmax ratio H reflex	No serious adverse effects
Yan, 2015	RCT	Cerebral Palsy	NI	E: 19 C: 20	17/22	E: 5.27 ± 1.91 C: 7.09 ± 3.05	rTMS + CR	Sham rTMS + CR	NI	E: 5 Hz/100%RMT C: 5 Hz/100%RMT	E: 20 min	NI	E: bilateral side C: bilateral side	E: 20 sessions C: 20 sessions	Upper limb and lower limb	MAS	No serious adverse effects
Yang, 2021	RCT	Stroke	NI	E: 10 C: 8	8/10	E: 53.40 ± 11.14 C: 57.63 ± 11.19	rTMS	CR	E: F8C	E: 1 Hz/90%MT	E: 24 min	E: 1,200 pulses	E: unaffected side	E: 40 sessions	Upper limb	MAS	NI
Yang and Yang, 2022	RCT	Spinal Cord Injury	E: 5.82 ± 1.68 C: 5.43 ± 1.12	E: 89 C: 89	52/126	E: 36.25 ± 6.12 C: 35.39 ± 4.84	rTMS + CR	CR	E: F8C	E: 10 Hz/80%-90%RMT	NI	E: 620 pulses	NI	E: 50 sessions	Lower limb	MAS	NI
Yuan, 2020	RCT	Stroke	NI	E: 23 C: 10	9/24	E: 56.61 ± 11.84 C: 59.90 ± 10.51	rTMS + CR	CR	E: F8C	E: 1 Hz/90%RMT	E: 24 min	E: 1,200 pulses	E: unaffected side	E: 40 sessions	Upper limb	MAS	NI
Zang et al., 2022	RCT	Cerebral Palsy	NI	E: 40 C: 40	38/42	E: 3.06 ± 0.28 C: 2.97 ± 0.25	rTMS + CR	CR	NI	E: 5 Hz/100%MT	E: 20 min	NI	E: bilateral side	E: 20 sessions	Lower limb	MAS	NI
Zhang et al., 2020	RCT	Stroke	E: 1.61 ± 0.42 C: 1.67 ± 0.45	E: 40 C: 40	35/45	E: 50.14 ± 11.24 C: 52.41 ± 12.49	rTMS + CR	CR	NI	E: 1 Hz/90%RMT	NI	E: 1,500 pulses	NI	E: 10 sessions	Upper limb	MAS Hmax/Mmax ratio	NI
Zhang, 2018	RCT	Cerebral Palsy	NI	E1: 15 E2: 15 C: 15	16/29	E1: 4.77 ± 0.76 E2: 4.85 ± 0.72 C: 4.89 ± 0.73	rTMS + CR	Sham rTMS + CR	NI	E1: 1 Hz/90%MT E2: 5 Hz/90%MT C: 5 Hz/90%MT	E1: 20 min E2: 20 min	E1: 1,200 pulses E2: 1,200 pulses	E1: unaffected side E2: affected side	E1: 15 sessions E2: 15 sessions C: 15 sessions	Upper limb	MAS	NI
Zhao, 2021	RCT	Stroke	E: 2.87 ± 0.82 C: 2.81 ± 0.79	E: 50 C: 50	29/71	E: 56.32 ± 7.83 C: 56.29 ± 7.88	rTMS + CR	Sham rTMS + CR	E: double cone coil	E: 1 Hz/80%RMT	NI	E: 1,200 pulses	E: unaffected side C: unaffected side	E: 24 sessions C: 24 sessions	Upper limb	MAS	NI

RCT, randomized controlled trial; E1, low-frequency rTMS group; E2, high-frequency rTMS group; E3, low-frequency rTMS plus high-frequency rTMS group; C, control group; E, experimental group; rTMS, repetitive transcranial magnetic stimulation; CR, conventional rehabilitation; F8C, figure-of-eight coil; RMT, resting motor threshold; MAS, Modified Ashworth Scale; NI, no information; MT, motor threshold; Hmax/Mmax ratio, ratio of maximum H reflex to maximum M response.

### Risk of bias assessment

**FIGURE 2:**
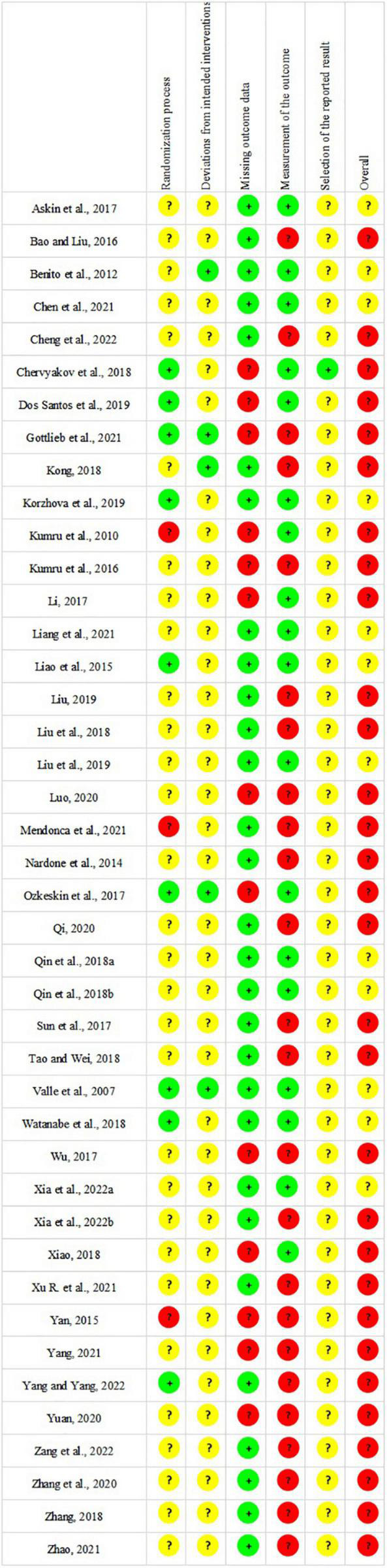
Risk of bias graph.

The ICC of each domain varied from 0.77 to 0.83, which indicated good reliability within risk of bias assessment. The results of risk of bias assessment are shown in Figure 2. Twenty-three RCTs (Valle et al., 2007; Liao et al., 2015; Yan, 2015; Bao and Liu, 2016; Askin et al., 2017; Li, 2017; Wu, 2017; Liu et al., 2018; Qin et al., 2018a,b; Tao and Wei, 2018; Xiao, 2018; Zhang, 2018; Liu et al., 2019; Qi, 2020; Chen et al., 2021; Gottlieb et al., 2021; Liang et al., 2021; Mendonca et al., 2021; Zhao, 2021; Xia et al., 2022b; Yang and Yang, 2022; Zang et al., 2022) adequately described methods of random sequences generation. Allocation concealment was performed in six studies (Ozkeskin et al., 2017; Chervyakov et al., 2018; Watanabe et al., 2018; Dos Santos et al., 2019; Korzhova et al., 2019; Mendonca et al., 2021), whereas the remaining studies did not report allocation concealment. Twelve studies (Valle et al., 2007; Kumru et al., 2010, 2016; Benito et al., 2012; Nardone et al., 2014; Liao et al., 2015; Li, 2017; Ozkeskin et al., 2017; Kong, 2018; Dos Santos et al., 2019; Korzhova et al., 2019; Mendonca et al., 2021) specified the blinding of patients and outcome assessors, and seventeen studies (Valle et al., 2007; Liao et al., 2015; Askin et al., 2017; Li, 2017; Ozkeskin et al., 2017; Chervyakov et al., 2018; Liu et al., 2018; Qin et al., 2018a,b; Watanabe et al., 2018; Xiao, 2018; Dos Santos et al., 2019; Korzhova et al., 2019; Liu et al., 2019; Chen et al., 2021; Liang et al., 2021; Xia et al., 2022a) mentioned the blinding of outcome assessors, while the rest of studies did not address whether blinding was used. In summary, the overall risk of bias of thirty studies (Kumru et al., 2010, 2016; Nardone et al., 2014; Yan, 2015; Bao and Liu, 2016; Li, 2017; Ozkeskin et al., 2017; Sun et al., 2017; Wu, 2017; Chervyakov et al., 2018; Kong, 2018; Liu et al., 2018; Tao and Wei, 2018; Xiao, 2018; Zhang, 2018; Dos Santos et al., 2019; Liu, 2019; Luo, 2020; Qi, 2020; Yuan, 2020; Zhang et al., 2020; Gottlieb et al., 2021; Mendonca et al., 2021; Xu R. et al., 2021; Yang, 2021; Zhao, 2021; Cheng et al., 2022; Xia et al., 2022b; Yang and Yang, 2022; Zang et al., 2022) was rated as “high risk of bias” and twelve studies (Valle et al., 2007; Benito et al., 2012; Liao et al., 2015; Askin et al., 2017; Qin et al., 2018a,b; Watanabe et al., 2018; Korzhova et al., 2019; Liu et al., 2019; Chen et al., 2021; Liang et al., 2021; Xia et al., 2022a) were considered as “some concerns.”

### Primary outcome-the modified Ashworth scale

A total of 42 (Valle et al., 2007; Kumru et al., 2010, 2016; Benito et al., 2012; Nardone et al., 2014; Liao et al., 2015; Yan, 2015; Bao and Liu, 2016; Askin et al., 2017; Li, 2017; Ozkeskin et al., 2017; Sun et al., 2017; Wu, 2017; Chervyakov et al., 2018; Kong, 2018; Liu et al., 2018; Qin et al., 2018a,b; Tao and Wei, 2018; Watanabe et al., 2018; Xiao, 2018; Zhang, 2018; Dos Santos et al., 2019; Korzhova et al., 2019; Liu, 2019; Liu et al., 2019; Luo, 2020; Qi, 2020; Yuan, 2020; Zhang et al., 2020; Chen et al., 2021; Gottlieb et al., 2021; Liang et al., 2021; Mendonca et al., 2021; Xu R. et al., 2021; Yang, 2021; Zhao, 2021; Cheng et al., 2022; Xia et al., 2022a,b; Yang and Yang, 2022; Zang et al., 2022) studies reported the scores of MAS. However, the results of the MAS in five studies could not be extracted (Benito et al., 2012; Watanabe et al., 2018; Dos Santos et al., 2019; Mendonca et al., 2021; Zang et al., 2022), and one study did not provide the results of the MAS in control group (Chervyakov et al., 2018).

#### Repetitive transcranial magnetic stimulation plus conventional rehabilitation versus sham repetitive transcranial magnetic stimulation plus conventional rehabilitation

**FIGURE 3:**
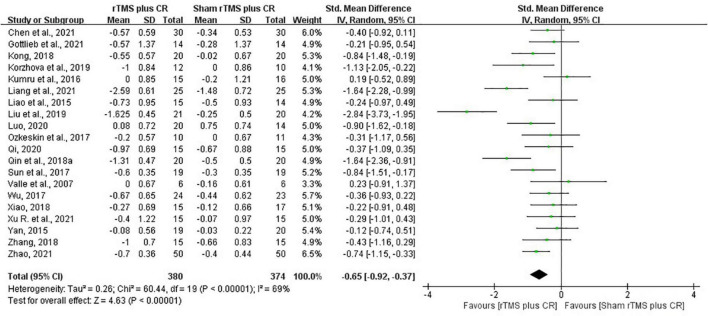
The forest plot of MAS in comparison of rTMS plus CR versus sham rTMS plus CR.

**FIGURE 4:**
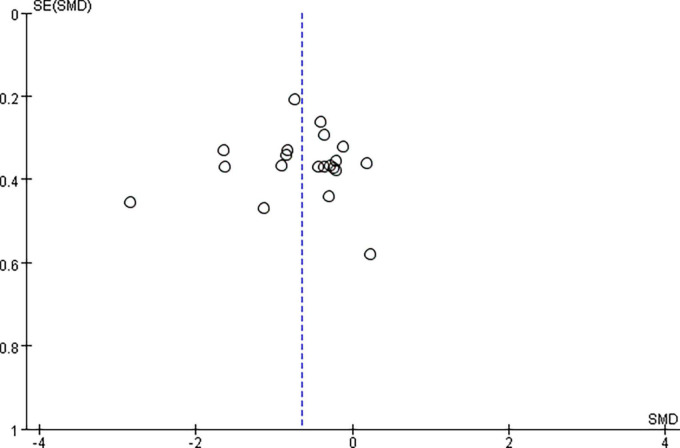
The funnel plot of MAS in comparison of rTMS plus CR versus sham rTMS plus CR.

Pooled data from the twenty RCTs (Valle et al., 2007; Liao et al., 2015; Yan, 2015; Kumru et al., 2016; Ozkeskin et al., 2017; Sun et al., 2017; Wu, 2017; Kong, 2018; Qin et al., 2018b; Xiao, 2018; Zhang, 2018; Korzhova et al., 2019; Liu et al., 2019; Luo, 2020; Qi, 2020; Chen et al., 2021; Gottlieb et al., 2021; Liang et al., 2021; Xu R. et al., 2021; Zhao, 2021) revealed that rTMS plus CR decreased more MAS scores than sham rTMS plus CR (SMD = –0.65, 95%CI = –0.92 to –0.37, *I*^2^ = 69%, *P* < 0.00001) (Figure 3). The funnel plot, *Egger’s* test (*P* = 0.764) and *Begg’s* test (*P* = 0.922), of the MAS scores indicated no publication bias (Figure 4).

#### Repetitive transcranial magnetic stimulation plus conventional rehabilitation versus conventional rehabilitation

**FIGURE 5:**
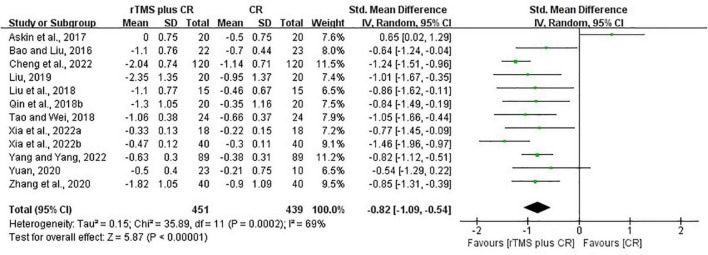
The forest plot of MAS in comparison of rTMS plus CR versus CR.

**FIGURE 6:**
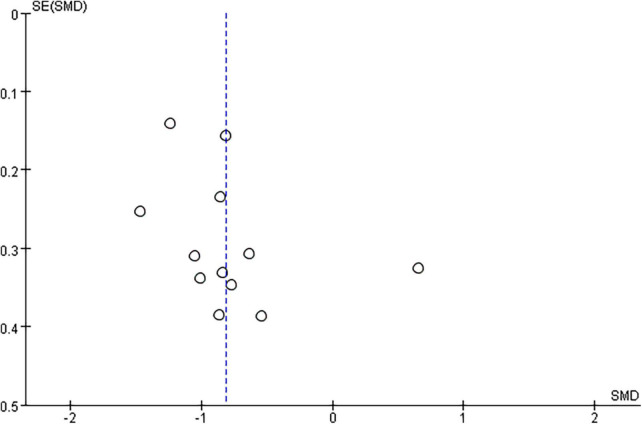
The funnel plot of MAS in comparison of rTMS plus CR versus CR.

Based on the data of 12 RCTs (Bao and Liu, 2016; Askin et al., 2017; Liu et al., 2018; Qin et al., 2018a; Tao and Wei, 2018; Liu, 2019; Yuan, 2020; Zhang et al., 2020; Cheng et al., 2022; Xia et al., 2022a,b; Yang and Yang, 2022), we found that rTMS plus CR could reduce more MAS scores than CR (SMD = –0.82, 95%CI = –1.09 to –0.54, *I*^2^ = 69%, *P* < 0.00001) (Figure 5). The funnel plot, *Egger’s* test (*P* = 0.192) and *Begg’s* test (*P* = 0.304), demonstrated that there was no publication bias (Figure 6).

#### Repetitive transcranial magnetic stimulation versus sham repetitive transcranial magnetic stimulation

**FIGURE 7:**
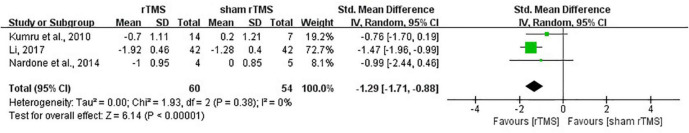
The forest plot of MAS in comparison of rTMS versus sham rTMS.

The results showed that rTMS was superior to sham rTMS in reduction of MAS scores according to the data from three studies (Kumru et al., 2010; Nardone et al., 2014; Li, 2017) (SMD = –1.29, 95%CI = –1.71 to –0.88, *I*^2^ = 0%, *P* < 0.00001) (Figure 7). No publication bias was detected based on the *Egger’s* test (*P* = 0.449) and *Begg’s* test (*P* = 1.000).

#### Repetitive transcranial magnetic stimulation versus conventional rehabilitation

Yang (2021) reported that rTMS effectively lowered MAS scores when compared with CR group.

### Subgroup analysis of primary outcome

The results of subgroup analysis of MAS scores are presented in Table 2.

**TABLE 2:** The results of subgroup analysis.

	*n*	Effect size (95% Cl)	*P*-value	*I* ^2^	*P*-value
**The results of subgroup analysis of MAS in comparison of rTMS plus CR versus sham rTMS plus CR**					
**The type of UMN injury**					
Stroke	13	–0.73 (–1.05, –0.40)	0.0001	69%	<0.0001
CP	4	–0.23 (–0.61, 0.14)	0.75	0%	0.23
SCI	2	–0.73 (–2.52, 1.05)	0.0002	93%	0.42
MS	1	–1.13 (–2.05, –0.22)	–	–	0.02
**The frequency of rTMS**					
Low frequency (≤1)	10	–0.50 (–0.70, –0.30)	0.64	0%	<0.00001
High frequency (>1)	10	–0.89 (–1.43, –0.35)	<0.00001	82%	0.001
**The intensity of rTMS**					
≤90% MT	16	–0.68 (–1.02, –0.35)	<0.00001	73%	<0.0001
>90% MT	4	–0.51 (–0.91, –0.10)	0.24	29%	0.01
**The total sessions of rTMS**					
≤10	4	–0.40 (–0.80, –0.01)	0.39	1%	0.05
>10	15	–0.74 (–1.06, –0.41)	<0.00001	74%	<0.00001
**The assessment position of MAS**					
Upper limb	15	–0.65 (–0.96, –0.34)	<0.0001	68%	<0.0001
Lower limb	4	–0.74 (–1.58, 0.11)	0.001	81%	0.09
**The results of subgroup analysis of MAS in comparison of rTMS plus CR versus CR**					
**The type of UMN injury**					
Stroke	10	–0.83 (–1.17, –0.48)	<0.0001	74%	<0.00001
CP	1	–0.64 (–1.24, –0.04)	–	–	0.04
SCI	1	–0.82 (–1.12, –0.51)	–	–	<0.00001
**The frequency of rTMS**					
Low frequency (≤ 1)	9	–0.72 (–1.09, –0.36)	0.0002	74%	0.0001
High frequency (> 1)	3	–1.03 (–1.45, –0.60)	0.09	59%	<0.00001
**The intensity of rTMS**					
≤90% MT	10	–0.74 (–1.05, –0.42)	0.0009	68%	<0.00001
>90% MT	2	–1.20 (–1.46, –0.95)	0.53	0%	<0.00001
**The total sessions of rTMS**					
≤10	3	–0.30 (–1.18, 0.59)	0.0006	86%	0.51
>10	8	–1.01 (–1.22, –0.80)	0.21	28%	<0.00001
**The assessment position of MAS**					
Upper limb	9	–0.77 (–1.15, –0.40)	<0.0001	77%	<0.0001
Lower limb	1	–0.82 (–1.12, –0.51)	–	–	<0.00001

95% CI, 95% confidence interval; MAS, Modified Ashworth Scale; rTMS, repetitive transcranial magnetic stimulation; CR, conventional rehabilitation; UMN, upper motor neuron; CP, cerebral palsy; SCI, spinal cord injury; MS, multiple sclerosis; MT, motor threshold.

#### Repetitive transcranial magnetic stimulation plus conventional rehabilitation versus sham repetitive transcranial magnetic stimulation plus conventional rehabilitation

Compared with sham rTMS plus CR, rTMS plus CR was more effective in stroke and MS. Meanwhile, rTMS plus CR had better effect in upper limb. In the comparison of rTMS plus CR versus sham rTMS plus CR, rTMS with > 10 sessions decreased more MAS scores than rTMS ≤ 10 sessions.

#### Repetitive transcranial magnetic stimulation plus conventional rehabilitation versus conventional rehabilitation

Repetitive transcranial magnetic stimulation plus CR decreased more MAS scores than CR in spastic patients with stroke, SCI and CP. Moreover, rTMS with total sessions > 10 could decrease more MAS scores than rTMS with total sessions ≤ 10.

### Secondary outcomes

As shown in Table 3, rTMS plus CR could increase more FMA scores and BI scores than control group. However, there was no difference between rTMS plus CR or rTMS group and control group in improving H_max_/M_max_ ratio and F-wave latency.

**TABLE 3:** The results of secondary outcomes.

	*n*	Effect size (95% Cl)	*P*-value	*I* ^2^	*P*-value
**The results of secondary outcomes in comparison of rTMS plus CR versus sham rTMS plus CR**					
H_max_/M_max_ ration	2	0.34 (–0.39, 1.07)	<0.00001	99%	0.36
F-wave latency	2	–0.23 (–0.62, 0.16)	0.47	0%	0.25
FMA-UL	7	7.38 (5.89, 8.87)	0.36	9%	<0.00001
BI	7	6.83 (2.20, 11.46)	<0.00001	86%	0.004
**The results of secondary outcomes in comparison of rTMS plus CR versus CR**					
FMA-UL	7	4.38 (1.65, 7.10)	<0.00001	94%	0.002
FMA-LL	1	1.30 (0.18, 2.42)	–	–	0.02
BI	8	5.58 (2.28, 8.88)	<0.00001	89%	0.0009
**The results of secondary outcomes in comparison of rTMS versus sham rTMS**					
H_max_/M_max_ ration	3	–0.01 (–0.11,0.09)	0.97	0%	0.86

95% CI, 95% confidence interval; MAS, Modified Ashworth Scale; rTMS, repetitive transcranial magnetic stimulation; CR, conventional rehabilitation; Hmax/Mmax ratio, ratio of maximum H reflex to maximum M response; FMA-UL, Fugl-Meyer-Assessment of upper limb; BI, Barthel Index; FMA-LL, Fugl-Meyer-Assessment of lower limb.

There is only one study (Yang and Yang, 2022) reported that rTMS plus CR could effectively reduce HAMA and HAMD in contrast to the CR (*P* < 0.05).

### Adverse events

Among forty-two included studies, eleven studies (Valle et al., 2007; Yan, 2015; Askin et al., 2017; Li, 2017; Kong, 2018; Tao and Wei, 2018; Dos Santos et al., 2019; Qi, 2020; Chen et al., 2021; Mendonca et al., 2021; Xu R. et al., 2021) reported that all patients could tolerate rTMS without complications, and no serious adverse effects were occurred. Seven studies (Kumru et al., 2010, 2016; Benito et al., 2012; Luo, 2020; Gottlieb et al., 2021; Xia et al., 2022a,b) described that 33 patients complained of twitching facial muscles, headache, pain in contralateral hand, dizziness, neck pain, and mild drowsiness after the rTMS treatment. The rest studies did not mention any adverse effects during rTMS treatment.

### Sensitivity analysis

**FIGURE 8:**
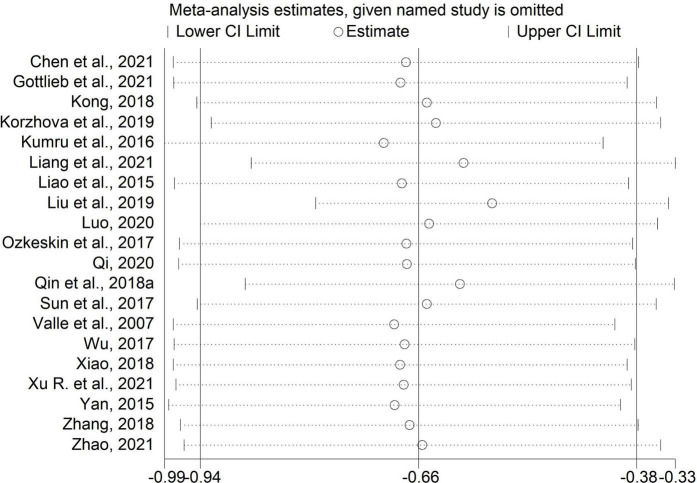
Sensitivity analysis for MAS (rTMS plus CR versus sham rTMS plus CR).

**FIGURE 9:**
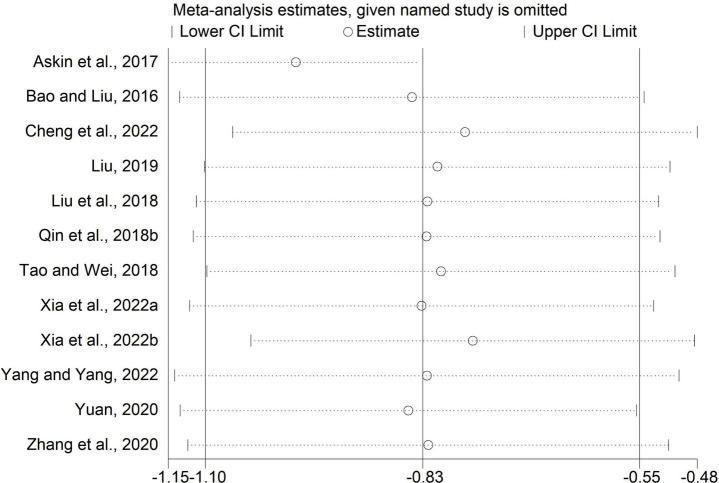
Sensitivity analysis for MAS (rTMS plus CR versus CR).

We performed sensitivity analysis by excluding one study each time. The results of the MAS scores in comparisons of rTMS plus CR versus sham rTMS plus CR and rTMS plus CR versus CR were unchanged (Figures 8, 9), which indicated these results were stable.

### Certainty of evidence

The ICC for the independent assessments of each item in the GRADE ranged from 0.81 to 0.85, which indicated satisfactory reliability. The certainty of evidence of each outcome was considered very low. The downgraded certainty of evidence was mainly caused by high risk of bias, and inconsistency of results. The results are shown in Table 4.

**TABLE 4:** The results of GRADE.

**Patient or population:** patients with spasticity after UMN injury **Settings:** **Intervention:** rTMS plus CR versus sham rTMS plus CR						
**Outcomes**	Illustrative comparative risks* (95% CI)		**Relative effect** **(95% CI)**	**No of Participants** **(studies)**	**Quality of the evidence** **(GRADE)**	**Comments**
	**Assumed risk**	**Corresponding risk**				
					
	**Control**	**rTMS plus CR versus sham rTMS plus CR**				

**rTMS plus CR versus sham rTMS plus CR**						
MAS		The mean MAS in the intervention groups was **0.65 standard deviations lower** (0.92–0.37 lower)		754 (20 studies)	⊕⊖⊖ **Very low**	⊖ SMD –0.65 (–0.92 to –0.37)
FMA		The mean FMA in the intervention groups was **0.89 standard deviations higher** (0.37–1.42 higher)		348 (7 studies)	⊕⊖⊖ **Very low**	⊖ SMD 0.89 (0.37 to 1.42)
MBI		The mean MBI in the intervention groups was **0.82 standard deviations higher** (0.23–1.4 higher)		358 (7 studies)	⊕⊖⊖ **Very low**	⊖ SMD 0.82 (0.23 to 1.4)
Hmax/Mmax		The mean Hmax/Mmax in the intervention groups was **0.34 higher** (0.39 lower to 1.07 higher)		90 (2 studies)	⊕⊖⊖ **Very low**	⊖ MD 0.34 (–0.39 to 1.07)
**F wave latency**		The mean f wave latency in the intervention groups was **0.23 standard deviations lower** (0.62 lower to 0.16 higher)		100 (2 studies)	⊕⊖⊖ **Very low**	⊖ SMD –0.23 (–0.62 to 0.16)
**rTMS plus CR versus CR**						
MAS		The mean MAS in the intervention groups was **0.82 standard deviations lower** (1.09–0.54 lower)		890 (12 studies)	⊕⊖⊖ **Very low**	⊖ SMD –0.82 (–1.09 to –0.54)
FMA		The mean FMA in the intervention groups was **0.98 standard deviations higher** (0.15 to 1.8 higher)		540 (8 studies)	⊕⊖⊖ **Very low**	⊖ SMD 0.98 (0.15 to 1.8)
MBI		The mean MBI in the intervention groups was **0.82 standard deviations higher** (0.24 to 1.39 higher)		547 (8 studies)	⊕⊖⊖ **Very low**	⊖ SMD 0.82 (0.24 to 1.39)
**rTMS versus sham rTMS**						
MAS		The mean MAS in the intervention groups was **1.29 standard deviations lower** (1.71 to 0.88 lower)		114 (3 studies)	⊕⊖⊖ **Very low**	⊖ SMD –1.29 (–1.71 to –0.88)
Hmax/Mmax		The mean Hmax/Mmax in the intervention groups was **0.01 lower** (0.11 lower to 0.09 higher)		52 (3 studies)	⊕⊖⊖ **Very low**	⊖ MD –0.01 (–0.11 to 0.09)
*The basis for the **assumed risk** (e.g., the median control group risk across studies) is provided in footnotes. The **corresponding risk** (and its 95% confidence interval) is based on the assumed risk in the comparison group and the **relative effect** of the intervention (and its 95% CI). **CI,** Confidence interval.						
GRADE Working Group grades of evidence **High quality:** Further research is very unlikely to change our confidence in the estimate of effect. **Moderate quality:** Further research is likely to have an important impact on our confidence in the estimate of effect and may change the estimate. **Low quality:** Further research is very likely to have an important impact on our confidence in the estimate of effect and is likely to change the estimate. **Very low quality:** We are very uncertain about the estimate.						

## Discussion

### The effect of repetitive transcranial magnetic stimulation for spasticity

Repetitive transcranial magnetic stimulation combined with CR or rTMS alone could effectively decrease MAS scores in spastic patients after UMN injury. According to the results of subgroup analysis, rTMS plus CR was more effective than control group in patients with stroke, which was consistent with previous systematic reviews (Graef et al., 2016; McIntyre et al., 2018). The minimum clinically important difference (MCID) refers to the smallest clinical change which is significant to patients (Stratford et al., 1998). Chen et al. (2019) reported that the MCID of MAS in stroke patients, the MCID of MAS between 0.5 and 0.8 indicated moderate clinical effect, and the MCID greater than 0.8 meant high clinical effect. In our study, the SMD of MAS in stroke patients (rTMS plus CR versus sham rTMS plus CR) was 0.73, and the SMD of MAS (rTMS plus CR versus CR) was 0.83. These results demonstrated that rTMS plus CR had a moderate-to-high clinical effect to relieve spasticity in stroke patients.

With regard to other types of UMN injury, we found that the results of rTMS for SCI, CP were inconsistent in different comparisons. Moreover, there was only one study focusing on rTMS for MS. Due to limited studies and no MCID of MAS in patients with SCI, CP, MS, these results warranted further investigation.

Furthermore, it is reported that excitatory neurotransmitter and inhibitory neurotransmitter play an important role in the pathogenesis of spasticity (Liu et al., 2021). In mouse model of middle cerebral artery occlusion (MCAO), the concentration of excitatory neurotransmitter Glutamate (Glu) increased in the ischemic area of cerebral hippocampus (Qian et al., 2022). Sun et al. (2022) found that the expression of inhibitory neurotransmitter Gamma-aminobutyric acid (GABA) in mouse model of MCAO decreased in the brainstem. Currently, Poh et al. (2019) observed that the concentration of Glu in C57BL/6J mouse brain reduced after rTMS treatment. Peng et al. (2021) discovered that rTMS with low frequency was able to increase GABA level in the central nervous system. Therefore, we speculated that the anti-spastic effect of rTMS may be associated with the decrease of excitatory neurotransmitters and the increase of inhibitory neurotransmitters. However, the mechanism of rTMS for spasticity is still unclear and needs to be further studied.

### The effect of repetitive transcranial magnetic stimulation for spasticity with different parameters

#### Different frequencies

The results of subgroup analysis demonstrated that rTMS with high or low frequency could alleviate spasticity after UMN injury. rTMS with high frequency (>1 HZ) can produce motor cortex excitation, whereas rTMS with low frequency (≤1 HZ) may induce motor cortex inhibition (Fitzgerald et al., 2006; Corti et al., 2012; Rossini et al., 2015). Fisicaro et al. reported that the affected hemisphere would produce a reduced inhibition on the unaffected hemisphere after stroke (Fisicaro et al., 2019). For stroke patients, rTMS with high-frequency stimulation on unaffected hemisphere or low-frequency stimulation on affected hemisphere may regulate the excitability of cerebral cortex, restore the inter-hemispheric excitation/inhibition balance, ameliorate spasticity, and enhance motor function (Takeuchi et al., 2005).

Three included studies reported that rTMS with high frequency was used to treat spastic patients with SCI, whereas the results were inconsistent. Quartarone et al. (2005) discovered that rTMS with high frequency could increase cortical excitability, while Mendonca et al. (2021) assumed that the increased cortical excitability induced by rTMS was not sufficient enough to influence the spasticity in SCI patients.

Three studies applied high frequency and two studies used low frequency to alleviate spasticity in patients with CP, while the results were contradictory. Furthermore, only one study investigated the effect of rTMS with high frequency for spasticity in MS patients. Therefore, more rigorous designed RCTs are needed to determine the effect of rTMS with different frequencies for spastic patients after UMN injury.

#### Different sessions

According to subgroup analysis, the rTMS > 10 sessions had better effect than ≤ 10 sessions in decreasing spasticity. Previous studies reported that rTMS with over 10 sessions could reduce more MAS scores in patients with SCI (Nardone et al., 2015), stroke, MS (Gunduz et al., 2014) and CP (Gupta et al., 2016). The spasticity was ameliorated with the increase sessions of rTMS. Whereas the dosage-effect relationship of rTMS stimulation for UMN injury remains to be explored.

Apart from the stimulation parameters mentioned above, the demographic factors (e.g., age, gender, disease duration) may have impact on the effect of rTMS. Todd et al. (2010) discovered that the effect of the 6 Hz rTMS was greater in young adults than in old individuals. Brihmat et al. (2022) concluded that young patients usually had greater potential for inducing plasticity changes in response to rTMS than elder participants. Hanlon and McCalley (2022) found that gender maybe a critical influencing factor on the effect of rTMS, and they inferred that the reason may be related with gender difference in gray matter density and gyrification, proximity of the brain to the scalp and cortical excitability. Furthermore, Fitzgerald et al. (2016) reported that the response to rTMS was greater in patients with shorter duration of illness. Future researches could focus on the influence of demographic factors on the effect of rTMS for spasticity in UMN injury.

### The different assessment positions of the modified Ashworth scale

The present systematic review included 33 studies focusing on upper limb, and six studies on lower limb, the results demonstrated that compared with sham rTMS plus CR, rTMS plus CR was effective to alleviate spasticity of upper limb, while uneffective for lower limb. Lin et al. (2015) observed that most of studies investigated the effect of rTMS on motor dysfunction of upper extremity after stroke, but few studies paid attention to lower extremity. The reason maybe that the motor areas of lower limb is located in the deep inter-hemisphere fissure, and it is difficult for rTMS to deliver stimulation (Kakuda et al., 2013; Foerster et al., 2018).

### The effect of repetitive transcranial magnetic stimulation for motor function and the activity of daily life

The results revealed that rTMS was effective to improve motor function and the activity of daily life. Li et al. (2022) reported that rTMS could dilate the cerebral blood vessels, increase the blood flow of brain tissue, and promote the regeneration of damaged axons, thus promoting the recovery of motor function (Sander et al., 1995; Wassermann and Lisanby, 2001). Previous studies also confirmed that rTMS could ameliorate muscle spasticity, improve motor function and the activity of daily life (Li D. et al., 2021; Kan et al., 2022).

### The strength and limitations of this study

This is the latest systematic review and meta-analysis which focused on the effects of rTMS for UMN injury. Additionally, we conducted comprehensive search and assessed the risk of bias with ROB2.0. This systematic review and meta-analysis was conducted and reported strictly following the AMSTAR 2.0 and PRISMA 2020 statement guidelines. However, the present study has some limitations. First, MAS was used to evaluate spasticity among included studies, which is too subjective to accurately reflect the change of spasticity. Therefore, the objective indicators (e.g., Hmax/Mmax ratio, F-wave latency) of spasticity should be applied in future studies. Second, most of included studies did not comprehensively evaluate the effect of rTMS for spastic patients after UMN injury. Future studies should comprehensively assess the general health status, mood changes and quality of life of spastic patients after UMN injury. Third, owing to limited studies, we could not determine the optimal stimulation protocols of rTMS on spasticity after UMN injury (the optimal time of rTMS treatment, the optimal intensity, frequency, et al.). The optimal stimulation protocols of rTMS for spastic patients after UMN injury remain for further exploration. Last, there were comparisons of rTMS plus CR versus sham rTMS plus CR, rTMS plus CR versus CR, rTMS versus sham rTMS, and rTMS versus CR in this systematic review and meta-analysis, the researchers should pay attention to the effect of rTMS in contrast to other active interventions (tDCS, oral muscle relaxants, botulinum neurotoxin injections, et al.).

## Conclusion

Repetitive transcranial magnetic stimulation could be recommended as an effective and safe therapy to relieve spasticity in patients with UMN injury. However, due to high heterogeneity and limited RCTs, this conclusion should be treated with caution. More rigorous designed RCTs are needed to determine the optimal protocol of rTMS for spastic patients after UMN injury.

## Data availability statement

The original contributions presented in this study are included in the article/Supplementary material, further inquiries can be directed to the corresponding authors.

## Author contributions

JL, RJ, and ZZ conceived this study. All authors selected, extracted, assessed, and analyzed the data and revised the manuscript for intellectual content. JF, HF, and XX drafted the manuscript.
