# Supplementary material for: The effectiveness and safety of repetitive transcranial magnetic stimulation on spasticity after upper motor neuron injury: A systematic review and meta-analysis
Source: Front Neural Circuits. 2022 Nov 8;16:973561. doi: 10.3389/fncir.2022.973561 (PMC9679509; doi:10.3389/fncir.2022.973561)
Supplement: Supplementary file 2 [file Data_Sheet_2.PDF]

Search strategy

China National Knowledge Infrastructure Search strategy

(SU=痉挛 OR SU=挛缩 OR SU=僵硬 OR SU=强直 OR SU=高肌张力 OR SU=肌张力增高 OR SU=肌张力亢进 OR SU=高渗肌肉 OR SU=抽搐 OR SU=抽动 OR SU=颤动 OR SU=颤抖 OR SU=刚性 OR SU=抽筋) AND (SU=经颅磁刺激 OR SU=TMS OR SU=Transcranial Magnetic Stimulation)

the Chinese Science and Technology Periodical Database Search strategy

(M=痉挛 OR M=挛缩 OR M=僵硬 OR M=强直 OR M=高肌张力 OR M=肌张力增高 OR M=肌张力亢进 OR M=高渗肌肉 OR M=抽搐 OR M=抽动 OR M=颤动 OR M=颤抖 OR M=刚性 OR M=抽筋) AND (M=经颅磁刺激 OR M=TMS OR M=Transcranial Magnetic Stimulation)

Wanfang database Search strategy

(主题=痉挛 OR 主题=挛缩 OR 主题=僵硬 OR 主题=强直 OR 主题=高肌张力 OR 主题=肌张力增高 OR 主题=肌张力亢进 OR 主题=高渗肌肉 OR 主题=抽搐 OR 主题=抽动 OR 主题=颤动 OR 主题=颤抖 OR 主题=刚性 OR 主题=抽筋) AND (主题=经颅磁刺激 OR 主题=TMS OR 主题=Transcranial Magnetic Stimulation)

China Biology Medicine Search strategy

("痉挛"[加权:扩展] OR "挛缩"[加权:扩展] OR "僵硬"[加权:扩展] OR "强直"[加权:扩展] OR "高肌张力"[加权:扩展] OR "肌张力增高"[加权:扩展] OR "肌张力亢进"[加权:扩展] OR "高渗肌肉"[加权:扩展] OR "抽搐"[加权:扩展] OR "抽动"[加权:扩展] OR "颤抖"[加权:扩展] OR "刚性"[加权:扩展] OR "抽筋"[加权:扩展]) AND ("经颅磁刺激"[加权:扩展] OR "TMS"[加权:扩展] OR "Transcranial Magnetic Stimulation"[加权:扩展])

Pubmed Search strategy

#1 Transcranial Magnetic Stimulation[Mesh]

#2 Transcranial Magnetic Stimulation[tiab] OR Magnetic Stimulation, Transcranial[tiab] OR Magnetic Stimulations, Transcranial[tiab] OR Stimulation, Transcranial Magnetic[tiab] OR

Stimulations, Transcranial Magnetic[tiab] OR Transcranial Magnetic Stimulations[tiab] OR Transcranial Magnetic Stimulation, Single Pulse[tiab] OR Transcranial Magnetic Stimulation, Paired Puls[tiab] OR Transcranial Magnetic Stimulation, Repetitive[tiab] OR TMS[tiab] OR Repetitive Transcranial Magnetic Stimulation[tiab] OR rTMS

#3 #1 OR #2

#4 "Muscle Spasticity"[Mesh]

#5 hypertonus[tiab] OR spastic[tiab] OR tonus[tiab] OR cramp[tiab] OR dystonia[tiab] OR spasm*[tiab] OR tone[tiab] OR hypertonia*[tiab] OR hypertonicit*[tiab] OR rigidit*[tiab]

#6 #4 OR #5

#7 Randomized Controlled Trial[tiab] OR RCT[tiab]

#8 #3 AND #6 AND #7

Embase Search strategy

#1 (Transcranial Magnetic Stimulation) OR (Magnetic Stimulation, Transcranial) OR (Magnetic Stimulations, Transcranial) OR (Stimulation, Transcranial Magnetic) OR (Stimulations, Transcranial Magnetic) OR (Transcranial Magnetic Stimulations) OR (Transcranial Magnetic Stimulation, Single Pulse) OR (Transcranial Magnetic Stimulation, Paired Puls) OR (Transcranial Magnetic Stimulation, Repetitive) OR TMS

#2 hypertonus OR (muscle AND spasticity) OR spastic* OR tonus OR cramp OR dystonia OR spasm* OR tone OR hypertonia* OR hypertonicit* OR rigidit*

#3 Randomized Controlled Trial OR RCT

#4 #1 AND #2 AND #3

The Cochrane Library Search strategy

#1 (Transcranial Magnetic Stimulation OR Magnetic Stimulation, Transcranial OR Magnetic Stimulations, Transcranial OR Stimulation, Transcranial Magnetic OR Stimulations, Transcranial Magnetic OR Transcranial Magnetic Stimulations OR Transcranial Magnetic Stimulation, Single Pulse OR Transcranial Magnetic Stimulation, Paired Puls OR Transcranial Magnetic Stimulation, Repetitive OR TMS):ti,ab,kw

#2 (hypertonus OR Muscle Spasticity OR spastic* OR tonus OR cramp OR dystonia OR spasm* OR tone OR hypertonia* OR hypertonicit* OR rigidit*):ti,ab,kw

#3 (Randomized Controlled Trial OR RCT):ti,ab,kw

#4 #1 AND #2 AND #3

Web of Science Search strategy

(TS=Transcranial Magnetic Stimulation OR TS=Magnetic Stimulation, Transcranial OR TS=Magnetic Stimulations, Transcranial OR TS=Stimulation, Transcranial Magnetic OR TS=Stimulations, Transcranial Magnetic OR TS=Transcranial Magnetic Stimulations OR TS=Transcranial Magnetic Stimulation, Single Pulse OR TS=Transcranial Magnetic Stimulation, Paired Puls OR TS=Transcranial Magnetic Stimulation, Repetitive OR TS=TMS) AND (TS=hypertonus OR TS=Muscle Spasticity OR TS=spastic* OR TS=tonus OR TS=cramp OR TS=dystonia OR TS=spasm* OR TS=tone OR TS=hypertonia* OR TS=hypertonicit* OR TS=rigidit*) AND (TS=Randomized Controlled Trial OR TS=RCT)
