# Supplementary material for: The effectiveness and safety of repetitive transcranial magnetic stimulation on spasticity after upper motor neuron injury: A systematic review and meta-analysis
Source: Front Neural Circuits. 2022 Nov 8;16:973561. doi: 10.3389/fncir.2022.973561 (PMC9679509; doi:10.3389/fncir.2022.973561)
Supplement: Supplementary file 3 [file Data_Sheet_3.pdf]

No.	First Author and Year of publication	Title	Excluded Reason
1	Silvana 2014 (Barros et al., 2014)	Efficacy of Coupling Repetitive Transcranial Magnetic Stimulation and Physical Therapy to Reduce Upper-Limb Spasticity in Patients With Stroke: A Randomized Controlled Trial	Duplicate publication
2	Shen 2018 (Shen et al., 2018)	Effect of ultra-low frequency transcranial magnetic stimulation combined with rehabilitation training on motor function and speech function in children with cerebral palsy	Not RCT
3	Chen 2018 (Chen, 2018)	Effects of repetitive transcranial magnetic stimulation on spasm and motor function of lower limbs in patients with stroke	Not RCT
4	Dong 2016 (Dong, 2016)	Effect of Repetitive Transcranial Magnetic Stimulation on Spasticity in Patients with Incomplete Spinal Cord Injury	Not RCT
5	Jiang 2018 (Jiang et al., 2018)	Effect of Low Frequency Repetitive Transcranial Magnetic Stimulation Combined with Occupational Therapy on Recovery of Upper Limb Function in Stroke Patients	Not RCT
6	Kong 2019 (Kong, 2019)	Effects of repeated transcranial magnetic stimulation on spasticity in patients with incomplete spinal cord injury	Not RCT
7	Centonze 2007 (Centonze et al., 2007)	Repetitive transcranial magnetic stimulation of the motor cortex ameliorates spasticity in multiple sclerosis	Not RCT
8	Ju 2016 (Ju and Mou, 2016)	The Effect of repetitive transcranial magnetic stimulation on spasticity and excitability of cerebral cortex and spinal cord after spinal cord injury	Conference Abstract
9	Xiang 2012 (Xiang and Huang, 2012)	The clinical application of rTMS combined with body-weight-supported treadmill training on patients with chronic incomplete spinal cord injury	Conference Abstract
10	Yan 2007 (Yan and Zhou, 2007)	Promoting effect of low-frequency repetitive transcranial magnetic stimulation on motor function rehabilitation of acute cerebral infarction	Conference Abstract

11	Feng 2012 (Feng et al., 2012)	Effect of KF-10 Ultra-low Frequency Transcranial Magnetic Stimulator on Intelligence and Motor Function of Children with Spastic Cerebral Palsy	Conference Abstract
12	Liao 2015 (Liao et al., 2015)	Effects of repetitive Transcranial Magnetic Stimulation Combined with Rehabilitation Exercises on Hypermyotonia after Cerebral Apoplexy and the Corresponding Surface Electromyography Alterations	Conference Abstract
13	Yan 2006 (Yan et al., 2006)	A preliminary study of transcranial magnetic stimulation promoting motor function rehabilitation in patients with acute cerebral infarction	Conference Abstract
14	Du 2012 (Du et al., 2012)	The effect of ultra-low frequency transcranial magnetic stimulator on neurotransmitters in children with spastic cerebral palsy	Conference Abstract
15	Yang 2015 (Yang et al., 2015b)	Bilateral hemisphere transcranial magnetic stimulation for upper limb dysfunction and spasticity after stroke	Data cannot be extracted
16	Yang 2015 (Yang et al., 2015a)	Effect of Repetitive Transcranial Magnetic Stimulation on the Recovery of Limb Function in Patients with Lower Limb Spasticity after Stroke	Data cannot be extracted
17	Du 2019 (Du and Zhu, 2019)	Therapeutic research of ultra-low frequency repetitive transcranial magnetic stimulation in the rehabilitation of gross motor function of spastic cerebral palsy	Data cannot be extracted
